# Man Enough to Care: Intersections of Masculinities, Care, and Aging

**DOI:** 10.1111/nhs.70315

**Published:** 2026-03-10

**Authors:** Mgr. Daniela Rendl

**Affiliations:** ^1^ Department of Sociology, Faculty of Social Studies Masaryk University Brno Czechia

**Keywords:** aging, care, embodiment at work, gender, healthcare system, masculinities, nursing

## Abstract

This study explores the intersection of masculinities, care, and aging through in‐depth interviews with 12 men employed in nursing in the Czech Republic. Using a qualitative design grounded in inductive grounded theory, data were transcribed verbatim and analyzed through thematic analysis in ATLAS.ti, following COREQ guidelines. The analysis identified two contrasting strategies of performing masculinity within a feminized profession: the adaptation of hegemonic masculinity through the incorporation of caring elements, and the re‐masculinization of care through relationality, emotional openness, and the rejection of dominance. The findings also show that physical strength operates as an ambivalent resource—granting younger men legitimacy and status while becoming a source of vulnerability with age. By conceptualizing care as a universal human skill rather than a gendered role, the study contributes to critical research on men and masculinities. It expands the framework of caring masculinities by integrating the perspective of aging. Men in nursing thus appear “man enough to care,” while their practices both reinforce and challenge the gender order.

## Key Points

- The number of men entering the nursing profession is increasing, raising questions about male caregiving. This study shows that care is not inherently tied to a specific gender role but represents a universal human skill.
- Men in nursing employ two contrasting strategies: one based on dominance, physical strength, and distancing from the feminine characteristics of the profession; the other on emotional sensitivity, relationality, and non‐dominant forms of masculinity.
- The intersection of aging and masculinity in the healthcare profession reveals both vulnerabilities and structural privileges, reinforcing the need to examine gender hierarchies in caregiving professions critically.

## 1 Introduction

Men are rarely associated with caregiving roles. This text presents findings from an analysis of 12 in‐depth interviews with men employed in nursing, thematizing the intersection of masculinities,^1^ caregiving, and aging within the organizational structures of the healthcare system. Men continue to be only marginally represented in nursing, a profession globally characterized by strong gender segregation. Despite ongoing efforts to diversify the workforce, women still constitute approximately 90% of nurses worldwide (World Health Organization 2021), and the proportion of men remains low across all regions (Boniol et al. 2019). The Czech Republic reflects this pattern even more sharply: men account for only around 3% of the nearly 90 000 nurses employed nationally, placing the country well below the already modest European average. Although numerically scarce, men in Czech nursing tend to occupy more specialized positions and earn higher average salaries than women—122% of the female average—indicating the persistence of gendered hierarchies within healthcare organizations (ČZSO 2024). The workforce is also highly homogeneous, with foreign nationals representing less than 2% of nurses (ÚZIS ČR 2025), and ethnic minority men forming an almost negligible subgroup. The ethnic composition makes the Czech context a particularly revealing site for examining how masculinities are negotiated within a feminized profession.

Research on men in nursing has consistently shown that masculinities are not fixed or universal but fluid, relational, and shaped by institutional and cultural contexts (R. Connell 1995; J. Hearn 2004; Connell and Messerschmidt 2005). Within feminized occupations, men often navigate tensions between dominant gender norms and the relational, emotional, and embodied demands of caregiving. Some studies describe strategies that align with hegemonic masculinity—emphasizing physical strength, technical competence, or leadership—while others document the emergence of more relational, emotionally open forms of masculinity associated with the concept of caring masculinities (Hanlon 2012; Scambor et al. 2014; Elliott 2016; de Boise and Hearn 2017). Care itself is increasingly conceptualized as a universal human capacity rather than an inherently feminine role (Fraser 1996), and men's accounts of caregiving often include empathy, sensitivity, tenderness, and respect for intimacy.

Aging adds an underexplored dimension to these dynamics. Physical capability is frequently perceived as a masculine asset in healthcare settings, yet it may become a source of vulnerability as men grow older. The embodied realities of aging—declining strength, increased fatigue, or changing physical confidence—intersect with gendered expectations of competence and resilience. How men negotiate these shifts within a profession that is both physically demanding and culturally feminized remains insufficiently understood, particularly in Central and Eastern Europe, where gender norms and labor histories differ from Western contexts.

This study addresses these gaps by examining how men employed in nursing in the Czech Republic construct and perform masculinity in relation to caregiving and aging. Drawing on in‐depth interviews with 12 men and an inductive qualitative design, the analysis explores the strategies through which men adapt, resist, or transform dominant gender norms in their everyday professional practice. By conceptualizing care as a universal human skill and integrating the perspective of aging into the framework of caring masculinities, the study contributes to critical scholarship on men and masculinities. It offers new insights into how men in a highly feminized profession negotiate identity, vulnerability, and professional adequacy, and how their practices simultaneously reinforce and challenge the gender order.

## 2 Theoretical Framework

Sociological research of men as caregivers and on aging men within the framework of Critical Studies of Men and Masculinities (CSMM) is a relatively recent scholarly development. CSMM understands masculinities as active, dynamic projects shaped by specific social and cultural contexts rather than biological determinants. Raewyn Connell conceptualizes masculinity as produced through gendered relations structured by the dominance of certain men over women and over men who do not embody the ideal of *hegemonic masculinity* (R. Connell 1995). Homogenic masculinity stands in contrast to *emphasized femininity*, which centers on compliance with this gendered hierarchy and accommodates men's interests (R. Connell 1995). Men and masculinities are therefore viewed as diverse, differentiated, and shifting, leading scholars to speak of multiple *masculinities* rather than a single, uniform category (Cross and Bagilhole 2002).

In analyzing masculine practices in nursing, I use the categories of dominant and non‐dominant masculinities, as I do not engage with Connell's central focus on legitimizing gender inequalities (Messerschmidt and Bridges 2024). Within nursing, these forms are expressed through specific performances and through framings of practice that emphasize power, control, and strength—attributes that symbolically recast nursing as a non‐feminine profession. Such cultural hierarchies are not fixed but continually renegotiated (Connell and Messerschmidt 2005; J. Hearn 2004).

The fluid and relational nature of masculinities allows attention to forms characterized by emotional sensitivity, attentiveness to others' needs, and supportive behavior. This perspective highlights how men perceive and express emotions, linking them to the conceptualized strength of male emotions (de Boise and Hearn 2017; Pulcini 2012). Non‐dominant masculinities are frequently associated with caring masculinities, a concept developed as an antithesis to dominant forms because care work requires men to adopt values that diverge from hegemonic masculinity (Prattes et al. 2025; Roberts and Prattes 2024; de Boise and Hearn 2017; Elliott 2016; Scambor et al. 2014; Hanlon 2012). Scholars emphasize that caring masculinities represent men's commitment to gender equality. However, they are not universally accessible or equally rewarded: “caring masculinities are not equally available to all men, nor are they equally rewarded” (Prattes 2022, 730). Masculinities—including caring masculinities—must therefore be understood as situated, unevenly distributed, and structurally conditioned. Attending to individuals' nuanced positions helps refine both the breadth and conceptual clarity of caring masculinities.

The concept of caring masculinities is relatively recent (Elliott 2016; Scambor et al. 2014) but draws inspiration from Nancy Fraser's idea of the *universal caregiver*, which highlights the value of care work and encourages men's involvement. Fraser argued that care should be recognized as a fundamentally human activity rather than a female obligation (Fraser 1996). Caring masculinities, therefore, represent forms of masculinity grounded in feminist care‐ethics values such as attentiveness, interdependence, shared responsibility, support, and empathy. As Elena Pulcini notes, care continues to be culturally associated with women, a belief that persists despite significant transformations in women's roles (Pulcini 2012). Karla Elliott further describes caring masculinities as a shift away from domination and aggression toward interdependence and care (Elliott 2016). Analytically, it is essential to recognize both pillars of the concept—*embracing care* and *rejecting domination*. Scholars caution against treating masculinities as merely a hybrid of hegemonic masculinity with caring elements (Prattes et al. 2025). Instead, caring masculinities should be understood as practices rooted in rejecting domination and embracing positive emotions, mutual dependence, and relationality as core values of care.

Most research on non‐dominant masculinities has focused primarily on fatherhood. Anna Tarrant's work shows that men can construct and perform masculinities through nurturing roles with their children (Tarrant 2018), and studies across diverse father groups highlighted men's increasing emotional presence and responsiveness (e.g., Doucet 2017; Rehel 2014; Wall 2014; Chesley 2011; Šmídová 2008). However, such involvement—emotional attentiveness, responsiveness, or vulnerability—does not automatically signal an emancipatory shift toward rejecting domination. Fathers often engage in child‐focused activities, such as playing or talking, without assuming broader childcare responsibilities (Forsberg 2007, 2009, 2010). Without critical reflection, research on caring masculinities risks obscuring persistent patriarchal structures and underlying power dynamics. Nonetheless, caring for one's own children offers an important pathway toward non‐dominant masculinities by providing practical caregiving experiences that build confidence and competence (Doucet 2017). These studies show that skilled care is a learnable human capacity that can align with non‐dominant forms of masculinity.

Elli Scambor et al. argue that caregiving masculinities extend beyond fatherhood to include emotional support, attentiveness, and relational care for friends, elders, and colleagues (Scambor et al. 2014, 151). Scambor frames caregiving as a universal human capacity and notes that men increasingly move beyond auxiliary roles to engage more equitably despite structural barriers: “Men's share of care remains an essential issue for gender equality and social development… paved with obstacles and rife with possibilities” (Scambor et al. 2024, 7). This perspective underscores the emotional openness and transformative potential of caregiving. Similarly, Miranda Leontowitsch highlights the significance of caregiving for aging men, enabling deeper relational engagement and reshaping traditional masculine identities (Leontowitsch 2022). These insights have shifted the focus from hierarchical models of care grounded in protectionism or dominance (Connell and Messerschmidt 2005; J. Hearn 2004) toward practices rooted in equality and relational sensitivity. This transformation is grounded in and developed through practice, aligning with the transformative potential of male emotions (de Boise and Hearn 2017; Pulcini 2012; Šmídová 2008).

Understanding the relationship between masculinities and care requires recognizing that care is always context‐specific; men who provide care cannot automatically be seen as actors of gender equality. Rikka Prattes emphasizes that care is not inherently emancipatory, as this requires a structurally grounded rejection of domination. She therefore conceptualizes caring masculinities as practices of care linked to active resistance against gendered, racial, class‐based, and colonial oppression (Prattes 2022).

Caring masculinities are thus framed as a normatively oriented counter‐project to gender hegemony rather than a simple variation of it (Roberts and Prattes 2024). The concept rests on reflecting masculine privilege, rejecting domination and aggression, and decoupling care from femininity—affirming care as a human, not female, norm (Roberts and Prattes 2024, 518). Such a conceptualization of care as a universal human quality builds on the notion of the *universal caregiver* (Fraser 1996) and calls for supportive structural conditions.

Care performed by men does not necessarily embody transformative or emancipatory potential for shifting the gender order, as research on fatherhood illustrates. Unequal conditions of caregiving can produce a normative ideal of the “caring man” that rests on these very inequalities (Prattes 2022, 728) and may obscure the hybridization of hegemonic masculinity with elements of care (Prattes et al. 2025, 515). Caring masculinities can thus reflect an emancipatory rejection of domination, but also an adaptation compatible with hegemonic norms. This tension has generated two main scholarly streams. Earlier studies highlight the emancipatory potential of men's caregiving, suggesting that entering “women's work” may disrupt the traditional link between masculinity and occupation (Evans and Frank 2003) and showing that men often wish to care but are constrained by cultural norms (O'Connor 2015). More recent research challenges the assumption that men's presence in nursing automatically alters gender relations (Kluczyńska 2021). Some scholars argue that masculinity itself operates as a form of capital that reproduces privilege (Huppatz and Goodwin 2013) or that emotional competence can function as a new mode of hegemony rather than its erosion (Cottingham 2017). These studies diverge less in empirical findings than in their interpretive frameworks.

Research on men in feminized professions broadly examines the strategies men use to distance themselves from feminine associations within such work (Simpson 2004; Stanley et al. 2016; Martsolf et al. 2023). Other studies show that, despite formal equality measures, men in nursing may still encounter structural discrimination with implications for health‐care quality (Gauci et al. 2023). Elwér et al. highlight horizontal gender segregation and the status loss experienced by men in caring professions (Elwér et al. 2012; see Lupton 2000). Across the literature, men in nursing are shown to face stigmatization and challenges to their masculinity, while also benefiting from hidden advantages such as smoother career advancement (e.g., Gauci et al. 2023).

The performance of masculinities in professional caregiving—still a relatively under‐researched area (Martsolf et al. 2023; Stanley et al. 2016; Simpson 2004)—is often described through strategies that distance men from feminine associations within the profession. They may differentiate themselves from subordinate female roles by drawing on institutional advantages (Hanlon 2024; Cross and Bagilhole 2002) or by framing nursing as hard, physical, and “heavy and sweaty” work to maintain a privileged position (Brown 2009; Simpson et al. 2014; R. W. Connell 1989).

Initiatives to attract more men to nursing respond to findings that men in female‐dominated occupations fear feminization and stigma. Studies show that men often re‐masculinize feminized professions by distancing themselves from subordinate female roles—either by drawing on institutional advantages (Hanlon 2024; Cross and Bagilhole 2002) or by framing nursing as heavy, physical work to minimize its non‐masculine associations (Simpson et al. 2014; Brown 2009; Lupton 2000). Other research describes reclaiming status through performing hegemonic masculinity, displaying toughness even at the expense of health, or eventually withdrawing. In Van Wees et al.'s study, men characterized male nurses using traits of hegemonic masculinity—“strong, leading, advisory, hard‐working, courageous, stress‐resistant, stable, pragmatic, straight‐to‐the‐point, easy, and independent”—and some portrayed men as more suitable or cost‐effective caregivers (Van Wees et al. 2024, 1667).

Efforts to re‐masculinize nursing at the individual level also require confronting stereotypes about men in the profession. The tension between feminized notions of care and dominant masculine ideals contributes to the low number of men entering nursing, and those who do often feel pressure to maintain a masculine identity (Hanlon 2024). Within this context, caring masculinities offer an emancipatory opportunity: they allow men to embrace caring practices that strengthen patient relationships and counter the persistent belief that men are less caring than women (Sundus and Younas 2020). Caring men frequently emphasize “connection”—relationships of trust, closeness, and dignity—thus enacting the pillar of *embracing care*. Some also view their work as rejecting power logics and adopting values of an “alternative” masculinity, even at the cost of lower status, thereby fulfilling the pillar of *rejecting domination* (Prattes 2022).

However, men in care occupations may not advance equality; instead, they can colonize and dominate the field by infiltrating or taking it over (Simpson 2004). Research on men in non‐traditional roles shows that men may feel comfortable adopting “female” discourses of care while simultaneously drawing on more privileged discourses to offset any minority disadvantage. Their token status often affords them enhanced leadership opportunities and a more career‐oriented position (Simpson 2004). It is therefore essential to analyze men in care work without assuming that increased emotional expressiveness automatically signals greater gender equality (de Boise and Hearn 2017).

Beyond caregiving stereotypes, men in nursing may also face disadvantages linked to aging. Because masculinity is closely tied to the body, the physical manifestations of aging can threaten masculine status (Calasanti and King 2005). Recent sociological work adopts a critical perspective on aging men (Hearn et al. 2021; Tarrant 2018; Tarrant and Watts 2014; Calasanti and King 2005; J. Hearn 1995), emphasizing the need to distinguish the contexts in which aging is addressed and noting the culturally ingrained denial of aging (Twigg 2004). Bodily changes—central to the aging process—disrupt the idealized corporeality of masculinity (Calasanti and King 2005). Gregorová and Šmídová highlight the emotional and bodily costs of maintaining hegemonic ideals, which affect even those who benefit from them most (Gregorová and Šmídová 2026). Studies show that masculinity is often defined in relation to femininity and homosexuality, through traits such as physical strength, dominance, leadership, and intellectual excellence (Clarke and Lefkowich 2018). Within this framework of masculine privilege, the denial of aging reveals structurally embedded fragility and vulnerability.

## 3 Methodology

The primary aim of this study is to map and understand the unique experiences of men in nursing, who in the Czech Republic constitute only 3% of the 90 000 nurses in the profession, and to situate these experiences within a broader societal context. The study focuses on men's capacity to provide care and the characteristics of such care in the Czech Republic. It draws on an analysis of interviews with 12 men aged 21–64 employed in nursing, all with at least higher professional education and working in 10 hospital wards in the Czech Republic. The study was reported in accordance with the COREQ guidelines (Tong et al. 2007).

### 3.1 Data Collection

The interviews were conducted as part of a broader research initiative examining masculinity, aging, power, and care in the Czech context—Institutions of Aging Men (IAM)—undertaken by an eight‐member team of sociological researchers. For this study, eight interviews were conducted by the lead author, and an additional four by the principal investigator of the IAM project (as acknowledged in the Acknowledgments). At the time of data collection, both the lead author and the colleague involved in co‐coding were doctoral researchers, whereas the project supervisor held a PhD. All members of the research team were employed as research fellows, had completed formal training in qualitative methodologies, and possessed prior experience with qualitative inquiry. Ongoing collaborative discussions within the wider IAM team supported the analytical process. All methodological details are reported in accordance with the COREQ guidelines (Tong et al. 2007).

Data were collected between 2023 and 2024 through in‐depth, semi‐structured interviews conducted in café settings (as decided by the research participants) to create a comfortable, confidential atmosphere (Wildemuth and Zhang 2009) and to support a respectful, conversational interaction (Kaufmann 2010). Interviews followed a qualitative design and were guided by key questions and thematic areas derived from relevant literature, while their course varied according to participants' preferences and communicative styles.

The informal yet confidential setting, environment, together with a dialogical tone, fostered openness, encouraged the emergence of new topics, and enabled flexible adaptation to participants' narratives. This approach also reduced hierarchical distance. Participants were approached as active co‐constructors of social reality (Kaufmann 2010) and as agents capable of contributing to “positive, ethical, communitarian change” (Denzin et al. 2006, 779).

The interview process was grounded in openness, reflexivity, and flexibility, informed by interpretive and critical paradigms (Lamnek and Krell 2016). These principles shaped data collection and analysis, and the triangulation with them, together with the generous time devoted to each interview, supported credibility in line with trustworthiness criteria (Lincoln and Guba 1985). Transferability was strengthened through rich contextual descriptions; dependability through multiple researchers' engagement with transcripts and the COREQ checklist; and confirmability through sustained reflexivity and emphasis on participants' own accounts.

By incorporating diverse positionalities and perspectives, the qualitative design also enabled attention to performativity, self‐reference, dramatization, and sensitivity to intimacy—dimensions essential for understanding the nuanced dynamics of masculinity, care, and aging. Interviewing men about their perception of masculinity presents methodological challenges, as traditional masculine norms may inhibit the expression of uncertainty or emotion (Davison 2007). Drawing on feminist approaches (Oakley 1998), particular attention was paid to the gendered and relational hierarchy between the researcher and the participants.

To foster openness, I selectively shared my professional background in nursing and occasional personal experiences, signaling receptivity to questioning traditional gender norms. This supported rapport, encouraged participants' willingness to share, and enabled reflexive awareness of my positionality as a woman interviewing men (J. Hearn 2013). Through the interviews, I reflected on the gender dynamics of the research relationship, listened actively, and used follow‐up questions to deepen understanding and avoid researcher‐driven interpretations (transferability). Participants were also encouraged to ask their own questions to disrupt traditional research hierarchies. Continuous reflexivity regarding power dynamics helped prevent misinterpretation (confirmability), in line with COREQ. Alongside the reflexive practice, I adopted a critical stance toward the topic and a commitment to supporting the recognition and emancipation of men within nursing.

### 3.2 Communication Partners

Recruiting participants was challenging due to the small number of men in Czech nursing—particularly in older age groups—and because some were hesitant to discuss their work experiences. Recruitment followed snowball sampling (Noy 2008). The first participant was contacted for his public advocacy for men in nursing; three responded to a call in a Facebook group; four were approached in retirement homes (three nursing assistants, one nurse); one was obtained based on a recommendation from the author of a master's thesis examining men in healthcare; two paramedics working in bedside care were added when referrals began to overlap; and the final participant was contacted based on a newspaper interview. Data saturation was considered recruitment and reported in line with COREQ (Tong et al. 2007). All participants were White—reflecting the limited racial diversity among men in Czech nursing—and were employed in large cities. Sociodemographic characteristics are summarized in Table A1 (Appendix A).

### 3.3 Data Analysis

The data analysis followed an inductive open‐coding approach consistent with grounded theory (Charmaz 2006). Interviews were transcribed verbatim, and repeated reading ensured alignment between recordings and transcripts, supporting credibility and dependability. All transcripts were imported into ATLAS.ti 24 and coded individually by the author and two IAM colleagues (as noted in the Acknowledgments). Coding was then extensively discussed within the team to achieve intercoder agreement.

Codes and themes were refined through interpretative discussions and existing coding frameworks into thematic clusters and cluster‐level themes. Throughout the process, emphasis was placed on presenting findings clearly and consistently, ensuring they faithfully reflected the data (confirmability) and could be meaningfully applied to other contexts (transferability). The author identified the main analytical themes—masculinity, rejection of dominance, adaptation, care, remasculinization, aging—and subthemes including emotional support, relational openness, embodiment, fragility, and vulnerability. Trustworthiness criteria (Lincoln and Guba 1985) were met throughout and reported in accordance with COREQ (Tong et al. 2007).

### 3.4 Ethical Considerations and Study Limitations

The Ethics Committee of Masaryk University (Directive No. 5/2015) granted ethical approval for the study as part of the broader project the Institution of Aging Men. The ethical framework was informed by feminist principles of relational ethics, care, and situated knowledge, emphasizing attentiveness to power dynamics and respect for lived experience.

Participants signed printed informed‐consent forms, which are securely archived. Consent was treated as ongoing; participants could pause, redirect, or withdraw at any time. None withdrew, as documented in COREQ. Brief informal conversations before and after interviews helped build rapport and reduce hierarchical distance. Confidentiality was ensured through pseudonymization and restricted access to transcripts. Reflexive attention to positionality and interpretive authority mitigated risks of reproducing power imbalances. The study was guided by a feminist ethic of care, acknowledging gendered inequalities in nursing and aiming to contribute to men's recognition and emancipation within the profession.

This study is grounded in qualitative research principles and therefore does not seek to generate generalizable claims about all men working in nursing. The self‐selected nature of the communication partners naturally limits the representativeness of the sample; nevertheless, the findings offer layered, finely detailed insights into a phenomenon that remains markedly underexplored in the Czech context. These insights also situate the study within broader scholarship on caring men and men in feminized professions. Ultimately, the empirical evidence presented here—challenging gendered assumptions about caregiving and highlighting the interplay between masculinity and aging—can meaningfully contribute to academic knowledge in these fields.

## 4 Findings

This chapter introduces the thematic areas explored in the two subsequent subchapters, each of which offers a detailed analysis of how men engage in caregiving practices. The first subchapter examines ideas about the representation and forms of care provided by men in nursing—Who is “man enough” for nursing? It is divided into two sections: Adaptation to Hegemonic Masculinity through Incorporation of Elements of Care, and Re‐masculinization of the Nursing Profession. The second subchapter focuses on the effects of aging on men working in nursing—When is an aging man enough for nursing? It is structured into three sections: Embodiment—Physical Strength, Denial, and Acknowledging Aging. Each of these analytical parts addresses both personal and institutional dimensions, shaped by broader structural and cultural conditions.

### 4.1 Who Is “Man Enough” for Nursing?

Nursing is a highly feminized profession in the Czech Republic—Men constitute less than 3%. However, they tend to attain higher levels of specialization and earn 122% of the average salary of women in the profession (ČZSO 2024). Individual workplaces, as well as the healthcare system as a whole, reflect the cultural hierarchies of a given society, in which the gender order shapes the nature of occupations (Harding 1986; Bourdieu 2001). Nurses and physicians exemplify a professional dichotomy in which medicine is associated with masculine traits and nursing with feminine dispositions (Bourdieu 2001; Parsons 1951).

Cultural hierarchies are not fixed; they are continually renegotiated (J. Hearn 2004; Connell and Messerschmidt 2005: 44). In this analysis, I show (a) how the nursing profession is changing with the increasing—albeit still modest—number of men entering it, and (b) how men in nursing describe their own transformation. Existing sociological studies of men as caregivers within the CSMM framework conceptualize masculinity as the outcome of relational dynamics within a specific gender order, grounded in the dominance of hegemonic masculinity and the subordination of emphasized femininity (R. Connell 1995).

This analysis approaches masculinities as diverse, differentiated, and fluid forms (Cross and Bagilhole 2002). It does not focus on the central point of Connell's conceptualization—namely, the legitimation of inequalities between men and women (Messerschmidt and Bridges 2024). Instead, it examines the performance of masculinities through dominance or participation. The experiences and evaluations presented here show that men's performances of masculinity in nursing oscillate between adapting to and disrupting the existing gender order of this feminized profession.

#### 4.1.1 Adapting Hegemonic Masculinity by Incorporating Elements of Care

The first quotation illustrates a participant's view of the ideal gender composition of a nursing team and his understanding of masculinity in nursing:

When at least a third of the team is men, it is much better. However, then again, it depends… This will sound bad, but not every man is a man. It really will sound bad, but if a man does not smell of sweat at the end of a shift, he is either slacking off—which he should not be, in my opinion—or he is a perfumed guy who does not belong here. Before coming here, I spent twenty minutes in the shower because there was no other way.

(Richard, 43 years old)

The participant considered a diverse team ideal, yet simultaneously distanced himself from men who did not fit his notion of masculinity. The expression “perfumed guy” suggests that he perceived such men as overly groomed—potentially associated with traits of *emphasized femininity* (R. Connell 1995). In other words, he viewed them as less entitled, weaker, less competitive, and less independent than what aligned with his image of a “real man.” By distancing themselves from the feminine characteristics associated with the profession (Simpson 2004; Stanley et al. 2016; Martsolf et al. 2023), men in nursing employ a strategy to preserve their masculine status and the privileges that accompany it.

Emphasizing physical strength and bodily odor as markers of hard work also serves to distance nursing from its feminine‐coded characteristics (Hanlon 2024; Cross and Bagilhole 2002; Simpson et al. 2014; Brown 2009; Connell and Messerschmidt 2005; R. Connell 1995). Such framing of the nursing profession corresponds to the fear of status loss described in the literature, a concern experienced and articulated by men working in caregiving professions (Elwér et al. 2012; see also Lupton 2000). The effort to hybridize hegemonic masculinity by incorporating elements of care (Prattes et al. 2025) can thus be understood, in these cases as well, as a response to this fear.

Another characteristic associated with masculinity appears in the following participant's response to what he values in his profession:

Precisely what I enjoy is the resuscitation, securing the patient, figuring out what is wrong (…) you never know what you are walking into (…) you have a few pieces of information (…) and it can all be completely different.

(Patrik, 24 years old)

This statement highlights action, unpredictability, and challenge—an orientation toward saving lives. The participant frames his profession as heroic, thereby reproducing the ideal of hegemonic masculinity.

The following excerpt shows another way this ideal is reproduced when describing the care he provides:

I am glad when I can help other people, not only at work. Even in everyday life, when someone needs advice or help, I am willing, and I am glad I can provide information or assistance. So that applies to work as well.

(David, 26 years old)

The participant emphasizes his ability to “advise” both within and outside the profession. Given his age, it is likely that he derives a certain status from his profession, enabling him to be valued within the social hierarchy.

The following passage illustrates how another participant evaluates his own ability to provide care:

Care, well, yeah, I can do it (…) Some guys have a much better feel for the work. I know some who are really meticulous, down to the details. I am not that meticulous. (…) Look, I do not mind caring; I do it, I do what I have to, but I am not a perfectionist or a meticulous person, so it does not look nice (…) I do it technically.

(Simon, 44 years old)

Here, care is associated with meticulousness, yet it is also accompanied by distancing from it. The participant evaluates his work as technical. This contrast can be interpreted as reflecting a broader societal dichotomy between caring practices and technical skills.

These quotations show that men in nursing do not necessarily represent a transformative or emancipatory potential for changing the gender order (Prattes 2022). Their accounts often frame nursing in opposition to feminine characteristics such as being “perfumed” or meticulous. Instead, they emphasize heroism, technicality, and the role of an adviser. This aligns with studies showing that men define their masculinity in relation to femininity and homosexuality primarily through physical strength, leadership, and intellectual excellence (Clarke and Lefkowich 2018)—that is, through dominance and control.

The mere presence of men in nursing does not inherently alter the gender order. On the contrary, it may reinforce it through the use of structurally conditioned privileges (Prattes 2022). In participants' accounts, masculine status is associated with hard physical work, bodily odor, heroism, or advisory roles—not with meticulousness.

Men's activity in nursing—similar to their involvement in childcare—does not necessarily imply a rejection of dominance. Fathers often engage only in certain aspects of childcare (Forsberg 2007, 2009, 2010). Likewise, the participants' accounts show a reframing of nursing through characteristics associated with masculinity.

This part of the analysis thus responds to the academic debate on whether care has a transformative or reproductive character. In other words, whether men's performance of care disrupts hegemonic masculinity (O'Connor 2015; Evans and Frank 2003) or, conversely, adapts it (Kluczyńska 2021; Cottingham 2017; Huppatz and Goodwin 2013).

The empirical accounts presented above show how care is reinterpreted to remain compatible with hegemonic masculinity—through emphasizing technicality, action, or heroism. In this sense, care becomes a space where masculinity is not only maintained but actively reaffirmed.

While the preceding section demonstrated how men in nursing may reproduce hegemonic masculinity through action‐orientation, technical competence, or distancing themselves from femininity, the following part of the analysis turns to the opposite pole of their experiences. It shows that the very same professional context can also serve as a site for the emergence of caring, non‐dominant forms of masculinity that unsettle the hegemonic model.

#### 4.1.2 Re‐Masculinization of the Nursing Profession

A different framing of what it means to provide competent care—namely, an understanding of care as a learnable human capacity—is illustrated in the following accounts:

The advantage in nursing is that when you provide care, you can choose where you want to work as a man, a woman, or anyone. We have entirely different demands for emotional intelligence, skills, and stress management among intensive care nurses (…) compared to long‐term care nurses.

(Oto, 47)

In this perspective, nursing is understood as a plural profession requiring diverse skills and value systems. Men within it need not embody the rigid power structures of hegemonic masculinity (J. Hearn 2001; R. Connell 1995). Care is thus not associated with sweat, technical prowess, or heroism, but with emotional intelligence, skill, and the capacity to manage stress.

A similar emphasis on emotional intelligence as an integral part of nursing appears in the following excerpt:

I started terribly naïve, raised in a way (…) that I must not allow myself any emotion, sadness, mistakes, and so on. And as life went on (…), I moved towards the psychological processes of a healthcare worker. (…) I am not saying I completely collapsed, but I stumbled, mentally, I think, and I sought psychological care, which helped me immensely.

(Theodor, 46)

The participant describes both his own transformation and the broader shift within nursing toward greater sensitivity to one's emotional experience. In doing so, he illustrates an inclusive, non‐dominant masculinity characterized by emotional intelligence, attentive listening, stress management, and responsiveness to others' needs (de Boise and Hearn 2017; Elliott 2016; Scambor et al. 2014; Hanlon 2012; Pulcini 2012). It further illustrates the transformative potential of male emotions (de Boise and Hearn 2017; Pulcini 2012; Šmídová 2008). He also speaks openly about his psychological difficulties and how he addressed them. In his view, nursing is a profession that requires men to cultivate skills and values that diverge from masculinity defined by dominance, strength, competitiveness, and independence (R. Connell 1995). Non‐dominant masculinities are frequently associated in the literature with caring masculinities, which require men to adopt values and dispositions of care that stand in tension with dominant masculinity (Hanlon 2012; Scambor et al. 2014; Elliott 2016; de Boise and Hearn 2017; Roberts and Prattes 2024; Prattes et al. 2025).

Non‐dominant, caring masculinities are closely tied to the acceptance of relationality and mutual dependence:

I worked in follow‐up care, a nice job, wound care, lots of those psychological aspects of care (…), so the key in caring for people was to care for good relationships and bonds. (…) And the burden of caring for others' needs must presuppose one basic thing, and that is caring for one's own needs.

(Oto, 47)

This emphasis on relational care corresponds to the transformative potential of caregiving. It enables deep engagement with relational dynamics and the reshaping of traditional masculine identities (Leontowitsch 2022). These accounts shift attention away from hierarchical models of care based on protectionism or dominance (J. Hearn 2004; Connell and Messerschmidt 2005) and toward practices rooted in equality, relational sensitivity, and attentiveness to one's own needs.

All preceding and subsequent quotations in this analytical subsection fully exemplify the two pillars of the concept of caring masculinities—*embracing care* and *rejecting dominance* (Prattes et al. 2025). They foreground attentiveness to others' needs, relationships, and emotional intelligence.

The following account offers a similar portrayal of care:

I would rather look after people, you know, the touchy‐feely stuff. Doctors do not really have that. They come in, solve some symptoms and (…) they see the problem, but they do not see the person as a whole (…). It is that healing, calming touch, like: you are not here alone, I will take care of you, everything is fine, nothing is happening, calm down.

(Quido, 34)

The participant describes care as human communication, engagement, and closeness through touch, as well as a holistic and empathetic approach. This closely aligns with how the literature conceptualizes the performance of masculinity through care, as involving emotional responsiveness and the provision of affect (de Boise and Hearn 2017), and as offering relief through tenderness, attentiveness, and care (Scambor et al. 2024).

In defining his role as a professional caregiver—particularly in contrast to the medical profession—the participant emphasized masculinity performed as attentiveness, comfort, and sensitivity. The masculinity he enacts bears no resemblance to dominance or to care as a form of control, where the caregiver directs and regulates those they care for. Instead, he provides care as an expression of non‐dominant masculinities (Hanlon 2012; Scambor et al. 2014; Elliott 2016; de Boise and Hearn 2017; Roberts and Prattes 2024; Prattes et al. 2025), characterized by openness to others' needs and a willingness to respond to them.

The final quotation in this section presents care as a way of recognizing the other as a unique human being, while also opening the important theme of intimacy:

I started noticing the patient's intimacy. I notice that every patient needs to eat, drink, be covered with a blanket, and have a hand held. (…) Because that person is not just a piece of meat that needs to stop bleeding.

(Theodor, 46)

This participant perceived sensitivity to intimacy as something that develops gradually through the practice of care itself, which aligns with conceptualizations of the transformative power of men's emotions (Pulcini 2012; de Boise and Hearn 2017). Men who provide care become more attuned to others' needs, enabling them to express tenderness, respond sensitively to others' intimacy, and offer relief through kindness and care. In professional nursing, men undergo training to become professional caregivers. Similarly, men who provide care within their families or social networks become caregivers through the practice of care (Scambor et al. 2024; Leontowitsch 2022; de Boise and Hearn 2017; Pulcini 2012).

This example demonstrates that men can embody social structures in ways that diverge from traditional expectations without disrupting their everyday practice of masculinity. The case of nursing shows that men can provide—and crucially, learn to provide—care, offer emotional support, and share human closeness (Scambor et al. 2024; de Boise and Hearn 2017). It also illustrates their capacity to recognize relational bonds with others (Leontowitsch 2022) and to enter interactions without dominance or manipulation (J. Hearn 2004; Connell and Messerschmidt 2005). Their performance of masculinity, although it may appear to depart from traditional expectations, is not perceived by them as problematic or as conflicting with their masculinity.

Men in nursing thus clearly illustrate care as a gender‐neutral concept that fulfills Fraser's (1996) idea of the “universal caregiver.” Care emerges as a fundamentally human capacity rather than a gendered role or biologically determined predisposition. The caring masculinities they perform constitute a normatively oriented counter‐project to gender hegemony (Roberts and Prattes 2024), enabling them to express openness and a sensitive engagement with the needs and experiences of those for whom they care.

### 4.2 When Is an Aging Man Enough for Nursing?

Nursing, as a physically and emotionally demanding profession, creates uncertainty—particularly for men—about whether they will continue to meet its requirements as they age. This section of the analysis first demonstrates how physical strength shapes men's experiences in nursing and then introduces denial and acceptance of aging as two distinct responses to the bodily changes associated with growing older.

#### 4.2.1 Embodiment—Physical Strength

This section of the analysis demonstrates that physical strength operates in nursing as an ambivalent source of masculinity: while for younger men it may function as a symbolic privilege and a means of legitimizing their presence in a feminized profession, with aging, the same expectation becomes a source of vulnerability and professional insecurity.

For men in nursing, the physically demanding nature of the profession may appear as an opportunity to construct a masculine identity associated with privilege and status through physical performance, yet it simultaneously represents a risk:

But I am not a forklift. I am not going to be flipping all the patients over for you.

(Vincent, 43 years old)

This expressive statement marks a distancing from established hospital practice, where patient handling is a significant component of nursing care, and where stereotypical gender expectations—assigning men greater physical strength—often lead to disproportionate demands.

As the following quotation shows, some men internalize this expectation:

I myself plan to take care of clients by lifting them, pulling them up, and checking their positioning.

(Cyril, 28 years old)

Some frame it as their advantage:

I don't want to underestimate women in this regard. I've realised through this work that they have more strength than people think. But I also know they're kind of glad to have a man in this position when they see someone who can help them, that they can save their backs or their bodies overall. So because I'm younger, maybe I have an advantage—my body is still… the strength is just there.

(David, 26 years old)

Also following statement describes similar expectations but adds a perspective that weakens the normative force of this demand:

It was explicitly emphasised that the girls need help lifting heavy things, turning patients, and those physically demanding tasks. (…) When there is a man, he usually carries the heavy stuff. But I do not think it's an obligation.

(Adam, 21 years old)

Accepting the expectation of greater physical strength—and performing it more frequently—aligns with broader masculine norms that link hard work and endurance to maintaining a privileged male position (R. W. Connell 1989; Brown 2009).

The final quotation in this thematic cluster further confirms the stereotypical demand for masculine performances of physical strength:

It is a bit of an exploitation of men in social services—they say he is stronger, (…) so we end up doing more heavy lifting than the women. (…) It is a kind of exploitation of that male strength.

(Cyril, 28 years old)

Gendered expectations that attribute greater physical strength to men provide certain advantages, yet they also compel men to enact forms of masculinity that emphasize physical power and fitness (R. Connell 1995; J. Hearn 2001). In the demanding context of nursing, physical labor is the most frequently mentioned aspect associated with aging. Masculinity is closely tied to the body, and age‐related bodily changes can threaten it (Calasanti and King 2005). Men in nursing may therefore encounter disadvantages not only due to stereotypes surrounding care work but also due to aging (Hearn & Parkin, 2021; Tarrant 2018; Tarrant and Watts 2014; Calasanti and King 2005; J. Hearn 1995). The expectation to maintain physical strength, combined with its decline in later life, generates fears of diminished professional performance—fears that appear more pronounced in men than in women. In this context, aging becomes a process of perceived loss, particularly the loss of strength.

#### 4.2.2 Denial

Younger men in the sample primarily emphasized expectations of physical strength, whereas older men who had begun to experience signs of aging adopted two distinct strategies: (a) denying symptoms and (b) openly acknowledging them.

An illustration of the first strategy appears in the following response to the question of whether he ever thought about his ageing or old age:

*No, no*.

(Gustav, 34 years old)

Given his age, it is possible that he does not yet perceive signs of aging; nevertheless, he was the only participant to avoid questions about age consistently. A clear example of a strategy for avoiding discussion of perceived aging—specifically the decline of physical strength—appears in the following excerpt:

I do not say that I am older. I say that I am more cautious. In the end, it is the same, but it sounds better, let's be honest.

(Richard, 43 years old)

This quotation shows that the participant was fully aware of replacing the word *‘older’ with ‘more cautious’*. Through this choice, he actively shapes how he is perceived and emphasizes agency rather than passivity. In line with traditional masculinities that prioritize activity, strength, and control (R. Connell 1995), he thus reframes aging as a personal, deliberate choice.

This strategy simultaneously reinforces his privileged position—after all, only someone who holds privilege can afford to be cautious. At the same time, it illustrates that masculinities are active, dynamic, and relational projects (R. Connell 1995; J. Hearn 2004). In this context, denying aging functions as a defense against threats to that position.

The oldest participant, already retired yet working alongside colleagues in a staff nurse position, spoke about aging only after repeated prompting. With evident reluctance, he explained that he left his managerial role due to:

health problems, so I needed time to recover.

(Ulrich, 64 years old)

When asked directly, he confirmed that he had taken medical leave and returned to a non‐managerial position. However, he denied that aging affected his professional performance in any way:

Look, when you're not in a managerial role, it doesn't limit you at all, really. I am above it.

(Ulrich, 64 years old)

Elsewhere in the interview, he described avoiding professional training sessions—another potential element of a denial strategy:

Hospital management wants us to attend, but I do not anymore. I am old, that is for the young ones. I am done with all those lectures and trainings.

(Ulrich, 64 years old)

Here again, we see a reframing of physiological limits as an active choice, which obscures any suggestion of insufficiency. This move simultaneously maintains an emphasis on activity, strength, and control (R. Connell 1995). This approach aligns with findings that men define masculinity in relation to femininity and homosexuality primarily through physical strength, dominance, and control (Clarke and Lefkowich 2018). It also confirms that bodily changes associated with aging can disrupt the idealized corporeality of masculinity (Calasanti and King 2005). By denying aging, they distance themselves from any form of subordination (Hanlon 2024). The signs of aging thus become a threat, and concealing them can be understood as an expression of fragility and vulnerability.

The physical demands of the profession offer opportunities to reinforce masculinities associated with strength and privilege (Martsolf et al. 2023; Stanley et al. 2016; Brown 2009; R. W. Connell 1989). At the same time, they carry risks, as corporeality is a key dimension of aging (Calasanti and King 2005; Twigg 2004).

#### 4.2.3 Acknowledging Aging

Other participants adopted a markedly different strategy. When asked whether he perceived signs of aging, one responded:

Of course, I managed things much better when I was younger. Now the physical fatigue is obvious. I get home, and we even joke about it—like an old uncle after the evening news, I make myself herbal tea, and I am in bed by nine at the latest.

(Theodor, 46 years old)

This quotation illustrates an open admission of declining physical capacity, framed with self‐deprecating humor. This participant works as a head nurse in a managerial role that no longer requires shift work. Later in the interview, he spoke candidly about the strain this places on him:

An ageing body regenerates much more slowly. Indeed, I have not been doing shifts for a long time; I am now used to a different rhythm. And when I occasionally take a shift—because I think it is important for a manager to know what is happening—I do it only because I want to. (…) But it is definitely much harder to recover from it.

(Theodor, 46 years old)

Another participant described similar difficulties:

Before, I had no problem switching from nights to days. Even if I slept only a little, I could function. Now it is worse—I am realising that sleeping during the day is absolutely not the same as sleeping at night (…) you are just constantly tired.

(Quido, 34 years old)

This openness is accompanied by explicit admiration for older female colleagues:

And I really admire some of the older women we have there, who still manage after 25 or 30 years. I really admire them, and they even work overtime more than I do (…) what I have to do is already my maximum.

(Quido, 34 years old)

For this participant, it is not difficult to compare himself with older female colleagues and express admiration for their endurance. This stance represents the opposite of denying aging as a distancing from subordination (Hanlon 2024). Instead, it exemplifies caring masculinity as a normatively oriented counter‐project to gender hegemony (Roberts and Prattes 2024). Here, a male professional caregiver expresses recognition and respect toward women, actively destabilizing his own gendered privileges. He enacts both pillars of caring masculinities—*embracing care* (reciprocity, compassion, dignity) and *rejecting domination* (Prattes 2022)—and thus contributes, at an individual level, to a re‐masculinization of nursing grounded in care rather than dominance.

## 5 Conclusion

This article, based on interviews conducted in the Czech Republic, argues that care holds far greater significance for men employed in nursing than conventional gender roles would suggest; that men are fully competent caregivers; and that gender hierarchy and aging simultaneously render men more vulnerable while also exposing the contours of their privilege. A closer examination of men's position within the nursing profession shows that their numerical underrepresentation does not necessarily imply exceptionalism or deviation, as has historically been the case for women entering male‐dominated fields (Gauci et al. 2023; Stanley et al. 2016; O'Connor 2015; Huppatz and Goodwin 2013; Riska 2011, 2001; Britton 2000; Fisher 1995).

The analysis reveals two contrasting strategies through which masculinities are performed in nursing: adaptation and re‐masculinization. The first strategy aligns masculinity with dominance (R. Connell 1995; Messerschmidt and Bridges 2024) and with traditionally masculine attributes (Hanlon 2024; Cross and Bagilhole 2002; Simpson et al. 2014; Brown 2009; Connell and Messerschmidt 2005). It can be understood as a response to fears of status loss (Elwér et al. 2012; see Lupton 2000) and as a hybridization of hegemonic masculinity through the incorporation of caring elements (Prattes et al. 2025). The findings show that men in nursing do not necessarily embody a transformative potential for the gender order (Prattes 2022); instead, they may reinforce it by distancing themselves from femininity and homosexuality through dominance and control (Clarke and Lefkowich 2018). In this context, care is reinterpreted to remain compatible with hegemonic masculinity—through an emphasis on physical labor, technical skill, heroism, and stress management (Forsberg 2007, 2009, 2010; Kluczyńska 2021; Cottingham 2017; Huppatz and Goodwin 2013). The mere presence of men in nursing may therefore stabilize, rather than disrupt, the existing gender order (Prattes 2022), a conclusion further supported by the findings of this study, which illustrate the hybridization of hegemonic masculinity through the incorporation of elements of care.

The second strategy represents the opposite pole of experience, presenting nursing as a space for cultivating caring, non‐dominant forms of masculinity. Here, men do not embody the rigid power structures of hegemonic masculinity (R. Connell 1995; J. Hearn 2001) but instead describe personal transformation—and a transformation of the profession—toward sensitivity, relationality, and emotional openness (Elliott 2016; Scambor et al. 2014; Hanlon 2012; Pulcini 2012; de Boise and Hearn 2017). Embracing the values of care, speaking openly about psychological difficulties, and rejecting dominance can be understood as fulfilling both pillars of the concept of caring masculinities—*embracing care* and *rejecting domination* (Prattes et al. 2025; Roberts and Prattes 2024; Leontowitsch 2022). In this framing, care appears as human communication, closeness, empathy, and a source of relief through tenderness and attentiveness (Scambor et al. 2024). Men in nursing thus illustrate care as a gender‐neutral concept aligned with Fraser's (1996) vision of the “universal caregiver”—a fundamentally human capacity rather than a gendered role. The findings of this study substantiate this claim by demonstrating multiple instances in which men provide care competently, appropriately, and without contradiction or tension.

The analysis of embodiment and physical strength further highlights its ambivalent role: while physical capability may serve younger men as symbolic privilege and a means of legitimizing their presence in a feminized profession, the same expectation becomes a source of vulnerability with age (R. Connell 1995; J. Hearn 2001; Hearn & Parkin, 2021; Tarrant 2018; Tarrant and Watts 2014; Calasanti and King 2005; J. Hearn 1995). Men respond in two ways: by denying aging and reframing it as an active choice consistent with traditional masculinities (R. Connell 1995; Hanlon 2024), or by openly acknowledging it, thereby challenging the idealized corporeality of masculinity (Calasanti and King 2005; Twigg 2004). The physical demands of the profession thus simultaneously reinforce masculinities associated with strength and privilege (Martsolf et al. 2023; Stanley et al. 2016; Brown 2009; R. W. Connell 1989) while also exposing their fragility, as the costs of sustaining hegemonic ideals are borne even by those who benefit from them most (Gregorová and Šmídová 2026).

By documenting the professional practice of care in nursing, this article contributes to critical studies of men and masculinities, particularly by demonstrating the unproblematic integration of masculinity and care in men's accounts of nursing work. It conceptualizes care as a universal human skill and expands the notion of caring masculinities through an analysis of masculine performances in professional caregiving that incorporates an aging perspective. Men who provide professional care thus show they are “man enough to care” and contribute to a positive, non‐problematic understanding of the relationship between masculinity and care.

## 6 Relevance for Clinical Practice

This study provides valuable insights for clinical practice by challenging gendered assumptions about caregiving and recognizing men's capacity to be empathetic, attentive, and skilled nurses. Understanding the diverse ways in which men perform masculinities in caregiving roles can foster more inclusive workplace cultures and support the development of training and mentorship programs that validate emotional sensitivity and relational competence as professional strengths.

By acknowledging the interplay between masculinity and aging, healthcare institutions can better address the specific vulnerabilities of male caregivers, promoting age‐inclusive and gender‐equitable strategies in staff development, retention, and leadership opportunities. Ultimately, this research calls for a shift in clinical environments to value care as a universal human practice, not defined by gender, and to create supportive structures that empower all professionals—regardless of gender—to thrive in caregiving roles.

## Author Contributions

**Mgr. Daniela Rendl:** conceptualization, investigation, funding acquisition, writing – original draft, methodology, validation, formal analysis, data curation, resources.

## Funding

This work was supported by the Grantová Agentura České Republiky/Czech Science Foundation (GAČR), project Institutions of Aging Men 23‐05047S.

## Ethics Statement

This study is a part of a larger research project called the Institution of Aging Men, which was approved by Research Ethics at Masaryk University (Directive No. 5/2015). No further ethical approval is required for this study—not applicable.

Prior to the commencement of each interview, I engaged in a brief informal dialogue with every communication partner with the aim of establishing rapport and fostering mutual trust. During this preliminary exchange, I disclosed my research positionality and the motivations underpinning my engagement with the topic. I also provided an overview of my previous education and professional experience in the field of nursing.

All communication partners were furnished with a printed copy of the informed consent form, meticulously prepared in accordance with established ethical guidelines. The document comprehensively outlined the nature and objectives of the research and explicitly detailed participants' rights. Particular emphasis was placed on the procedures implemented to ensure the anonymization and confidentiality of all audio recordings. Additionally, participants were informed of their unequivocal right to terminate the interview at any stage or to withdraw from the study entirely, without any adverse consequence (none of them felt the need to take advantage of this offer). The study was conducted in accordance with the ethical standards established by the university as well as the professional guidelines of the relevant academic discipline.

## Conflicts of Interest

The author declares no conflicts of interest.

## Data Availability

The data that support the findings of this study are available from the corresponding author upon reasonable request.

## Endnotes

^1^
Following R.W. R. Connell (1995), I use the plural “masculinities” to reflect the plurality within the category of men.

## Appendix A

**TABLE A1:** Sociodemographic characteristics of participants. This table provides an overview of the sociodemographic characteristics of the 12 male nurses who participated in the study, including pseudonym, age, nursing role, current occupation, level of education, and marital status.

Pseudonym	Age	Type of nursing	Occupation	Education	Marital status
Adam	21	Man in nursing	Practical nurse	Higher vocational school	Single
Cyril	28	Man in nursing	Nursing assistant	High school	In a relationship
David	26	Man in nursing	Nursing assistant	High school	In a relationship
Gustav	34	Man in nursing	Nursing assistant	High school	Single
Oto	47	Man in nursing	Specialist nurse	University	Single
Patrik	24	Man in nursing	Practical nurse	University	Single
Quido	34	Man in nursing	Practical nurse	University	Single
Richard	43	Man in nursing/paramedic	Practical nurse	University	Divorced/in a relationship
Simon	44	Man in nursing/paramedic	Practical nurse	Higher vocational school	Divorced/in a relationship
Theodor	46	Man in nursing	Head nurse	University	Registered partner
Ulrich	64	Man in nursing	Practical nurse, retiree, former head nurse	University	Divorced/in a relationship
Vincent	43	Man in nursing	Head nurse, university lecturer	University	Registered partner
